# Dicyclo­hexyl­ammonium thio­cyanate

**DOI:** 10.1107/S1600536807067773

**Published:** 2008-01-04

**Authors:** M. Khawar Rauf, Masahiro Ebihara, Imtiaz-ud-Din, Amin Badshah

**Affiliations:** a Department of Chemistry, Faculty of Engineering, Gifu University, Yanagido, Gifu 501-1193, Japan; b Department of Chemistry, Quaid-i-Azam University Islamabad, 45320-Pakistan

## Abstract

In the crystal structure of the title compound, C_12_H_24_N^+^·NCS^−^, the anions and cations are linked through N—H⋯N and N—H⋯S hydrogen bonds, resulting in a chain along the *a* axis.

## Related literature

For related literature, see: Ng (1992 ▶, 1993 ▶, 1995*a* ▶,*b* ▶).
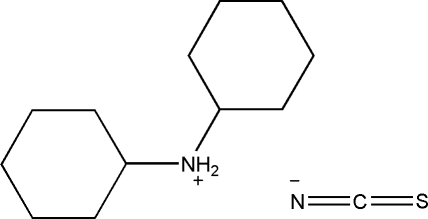


## Experimental

### 

#### Crystal data

C_12_H_24_N^+^·CNS^−^
*M* *_r_* = 240.40
Orthorhombic, 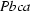

*a* = 8.781 (2) Å
*b* = 16.479 (4) Å
*c* = 19.026 (4) Å
*V* = 2753.2 (11) Å^3^
*Z* = 8
Mo *K*α radiation
μ = 0.21 mm^−1^
*T* = 123 (2) K
0.38 × 0.32 × 0.26 mm

#### Data collection

Rigaku/MSC Mercury CCD diffractometer
Absorption correction: none
20885 measured reflections
3151 independent reflections
3014 reflections with *I* > 2σ(*I*)
*R*_int_ = 0.029

#### Refinement

*R*[*F* ^2^ > 2σ(*F* ^2^)] = 0.041
*wR*(*F* ^2^) = 0.092
*S* = 1.20
3151 reflections
153 parameters
H atoms treated by a mixture of independent and constrained refinement
Δρ_max_ = 0.32 e Å^−3^
Δρ_min_ = −0.17 e Å^−3^

### 

Data collection: *CrystalClear* (Molecular Structure Corporation & Rigaku, 2001 ▶); cell refinement: *CrystalClear*; data reduction: *TEXSAN* (Rigaku/MSC, 2004 ▶); program(s) used to solve structure: *SIR97* (Altomare *et al.*, 1999 ▶); program(s) used to refine structure: *SHELXL97* (Sheldrick, 1997 ▶); molecular graphics: *ORTEPII* (Johnson, 1976 ▶); software used to prepare material for publication: *SHELXL97* and *TEXSAN*.

## Supplementary Material

Crystal structure: contains datablocks I, global. DOI: 10.1107/S1600536807067773/hg2363sup1.cif

Structure factors: contains datablocks I. DOI: 10.1107/S1600536807067773/hg2363Isup2.hkl

Additional supplementary materials:  crystallographic information; 3D view; checkCIF report

## Figures and Tables

**Table 1:** Hydrogen-bond geometry (Å, °)

*D*—H⋯*A*	*D*—H	H⋯*A*	*D*⋯*A*	*D*—H⋯*A*
N1—H1*B*⋯N2	0.901 (18)	1.986 (19)	2.8811 (17)	172.8 (16)
N1—H1*A*⋯S1^i^	0.926 (17)	2.440 (17)	3.3610 (13)	172.8 (13)

Symmetry code: (i) 
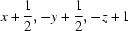
.

**Fig. 1.**
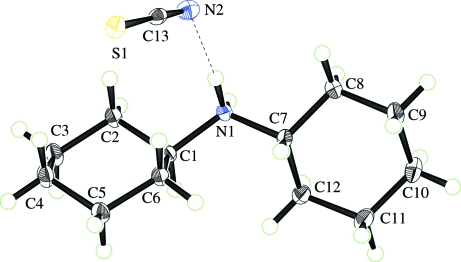
Molecular structure of (I) showing atom-labelling scheme and displacement ellipsoids at the 30% probability level.

**Fig. 2.**
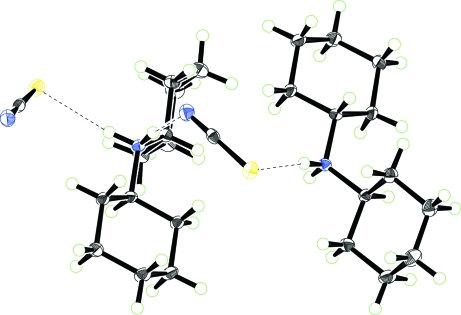
Showing hydrogen bonded anion to the cation through N—H···N and N—H···S.

## supplementary crystallographic information

### Comment

Ethanolic solution of dicyclohexylamine, when treated with equimolar amount of a dicarboxylic acid, affords the dicyclohexylammonium hydrogen dicarboxylate, which can be used in a condensation reaction with an organotin(IV) hydroxides or oxides to produce the corresponding organostannate (Ng, 1995*b*). The dicyclohexylammonium cation has been used in earlier studies to form crystalline derivatives of the dicarboxylic acids (Ng, 1992, 1993). The title compound (I) is an unexpected product of a reaction to synthesis a bifunctionalthiourea. As a result of the steric hindrance of the two cyclohexyl rings in the cation, the C—N—C angle is opened up to 117.23 (9)°, relative to the typical tetrahedral angle of 109.5°. Both of the cyclohexyl rings, exhibit chair conformations. The anionic thiocyanate group is strongly hydrogen bonded to the cation through N—H···N and N—H···S. All the other geometric parameters are in agreement with the previous studies of similar compounds (Ng, 1995*a*).

### Experimental

The title compound was obtained as an unexpected product from a reaction mixture containing dicyclhexylamine, benzoylchloride and potassiumthiocyanate in acetone, refluxed at 60 °C. Crystals were grown from a solution of the compound in toluene.

### Refinement

The nitrogen H atoms were refined isotropically. Other H atoms were placed in idealized positions and treated as riding atoms with C—H distance in the range 0.95–0.99 Å and *U*_iso_(H) = 1.2*U*_eq_(C) or 1.5*U*_eq_(C).

### Crystal data

**Table:**

C_12_H_24_N^+^·CNS^–^	*F*_000_ = 1056
*M**_r_* = 240.40	*D*_x_ = 1.160 Mg m^−^^3^
Orthorhombic, *P**b**c**a*	Mo *K*α radiation λ = 0.71070 Å
Hall symbol: -P 2ac 2ab	Cell parameters from 7454 reflections
*a* = 8.781 (2) Å	θ = 3.2–27.5º
*b* = 16.479 (4) Å	µ = 0.21 mm^−^^1^
*c* = 19.026 (4) Å	*T* = 123 (2) K
*V* = 2753.2 (11) Å^3^	Block, colorless
*Z* = 8	0.38 × 0.32 × 0.26 mm

### Data collection

**Table:**

Rigaku/MSC Mercury CCD diffractometer	3014 reflections with *I* > 2σ(*I*)
Monochromator: graphite	*R*_int_ = 0.029
Detector resolution: 14.62 pixels mm^-1^	θ_max_ = 27.5º
*T* = 123(2) K	θ_min_ = 3.2º
ω scans	*h* = −11→7
Absorption correction: none	*k* = −17→21
20885 measured reflections	*l* = −23→24
3151 independent reflections	

### Refinement

**Table:**

Refinement on *F*^2^	Secondary atom site location: difference Fourier map
Least-squares matrix: full	Hydrogen site location: inferred from neighbouring sites
*R*[*F*^2^ > 2σ(*F*^2^)] = 0.041	H atoms treated by a mixture of independent and constrained refinement
*wR*(*F*^2^) = 0.092	*w* = 1/[σ^2^(*F*_o_^2^) + (0.037*P*)^2^ + 1.0451*P*] where *P* = (*F*_o_^2^ + 2*F*_c_^2^)/3
*S* = 1.20	(Δ/σ)_max_ = 0.001
3151 reflections	Δρ_max_ = 0.32 e Å^−^^3^
153 parameters	Δρ_min_ = −0.17 e Å^−^^3^
Primary atom site location: structure-invariant direct methods	Extinction correction: none

### Special details

**Table:**

Geometry. All e.s.d.'s (except the e.s.d. in the dihedral angle between two l.s. planes) are estimated using the full covariance matrix. The cell e.s.d.'s are taken into account individually in the estimation of e.s.d.'s in distances, angles and torsion angles; correlations between e.s.d.'s in cell parameters are only used when they are defined by crystal symmetry. An approximate (isotropic) treatment of cell e.s.d.'s is used for estimating e.s.d.'s involving l.s. planes.
Refinement. Refinement of *F*^2^ against ALL reflections. The weighted *R*-factor *wR* and goodness of fit *S* are based on *F*^2^, conventional *R*-factors *R* are based on *F*, with *F* set to zero for negative *F*^2^. The threshold expression of *F*^2^ > σ(*F*^2^) is used only for calculating *R*-factors(gt) *etc*. and is not relevant to the choice of reflections for refinement. *R*-factors based on *F*^2^ are statistically about twice as large as those based on *F*, and *R*- factors based on ALL data will be even larger.

### Fractional atomic coordinates and isotropic or equivalent isotropic displacement parameters (Å^2^)

**Table:**

	*x*	*y*	*z*	*U*_iso_*/*U*_eq_	
N1	0.37002 (12)	0.15566 (6)	0.52333 (5)	0.0141 (2)	
H1A	0.4620 (19)	0.1381 (9)	0.5417 (8)	0.024 (4)*	
H1B	0.371 (2)	0.2103 (11)	0.5215 (9)	0.028 (4)*	
C1	0.36414 (14)	0.12612 (7)	0.44808 (6)	0.0145 (2)	
H1	0.3743	0.0657	0.4479	0.017*	
C2	0.49937 (14)	0.16257 (8)	0.40919 (6)	0.0176 (3)	
H2A	0.5952	0.1431	0.4308	0.021*	
H2B	0.4966	0.2224	0.4135	0.021*	
C3	0.49638 (15)	0.13897 (8)	0.33142 (7)	0.0209 (3)	
H3A	0.5820	0.1657	0.3067	0.025*	
H3B	0.5097	0.0795	0.3269	0.025*	
C4	0.34657 (15)	0.16400 (9)	0.29723 (7)	0.0220 (3)	
H4A	0.3372	0.2239	0.2980	0.026*	
H4B	0.3455	0.1461	0.2476	0.026*	
C5	0.21236 (15)	0.12627 (8)	0.33614 (7)	0.0208 (3)	
H5A	0.2169	0.0665	0.3314	0.025*	
H5B	0.1161	0.1451	0.3145	0.025*	
C6	0.21305 (14)	0.14887 (8)	0.41423 (6)	0.0173 (3)	
H6A	0.1958	0.2079	0.4193	0.021*	
H6B	0.1291	0.1202	0.4385	0.021*	
C7	0.24518 (14)	0.12773 (7)	0.57191 (6)	0.0151 (2)	
H7	0.1450	0.1455	0.5522	0.018*	
C8	0.26768 (15)	0.16798 (8)	0.64351 (6)	0.0184 (3)	
H8A	0.2641	0.2277	0.6382	0.022*	
H8B	0.3687	0.1531	0.6627	0.022*	
C9	0.14262 (17)	0.14035 (8)	0.69417 (7)	0.0239 (3)	
H9A	0.1596	0.1653	0.7408	0.029*	
H9B	0.0424	0.1588	0.6765	0.029*	
C10	0.14189 (17)	0.04800 (8)	0.70150 (7)	0.0263 (3)	
H10A	0.2385	0.0300	0.7233	0.032*	
H10B	0.0572	0.0314	0.7327	0.032*	
C11	0.12341 (16)	0.00738 (8)	0.62991 (7)	0.0241 (3)	
H11A	0.0214	0.0203	0.6108	0.029*	
H11B	0.1302	−0.0522	0.6357	0.029*	
C12	0.24540 (15)	0.03553 (7)	0.57788 (7)	0.0197 (3)	
H12A	0.3467	0.0168	0.5940	0.024*	
H12B	0.2255	0.0113	0.5312	0.024*	
N2	0.36405 (13)	0.32916 (7)	0.50487 (6)	0.0223 (2)	
C13	0.29457 (14)	0.35958 (7)	0.45967 (7)	0.0175 (3)	
S1	0.19380 (4)	0.40074 (2)	0.396108 (18)	0.02252 (11)	

### Atomic displacement parameters (Å^2^)

**Table:**

	*U*^11^	*U*^22^	*U*^33^	*U*^12^	*U*^13^	*U*^23^
N1	0.0148 (5)	0.0155 (5)	0.0121 (5)	0.0004 (4)	−0.0009 (4)	0.0003 (4)
C1	0.0159 (6)	0.0167 (5)	0.0108 (5)	−0.0004 (4)	−0.0004 (4)	−0.0018 (4)
C2	0.0130 (6)	0.0258 (6)	0.0141 (6)	−0.0001 (5)	0.0000 (5)	−0.0015 (5)
C3	0.0170 (6)	0.0309 (7)	0.0146 (6)	0.0008 (5)	0.0024 (5)	−0.0026 (5)
C4	0.0212 (7)	0.0322 (7)	0.0126 (6)	−0.0012 (5)	−0.0007 (5)	0.0018 (5)
C5	0.0176 (6)	0.0302 (7)	0.0145 (6)	−0.0033 (5)	−0.0034 (5)	−0.0002 (5)
C6	0.0134 (6)	0.0237 (6)	0.0147 (6)	−0.0020 (5)	−0.0001 (5)	−0.0008 (5)
C7	0.0144 (6)	0.0177 (5)	0.0132 (6)	−0.0002 (5)	0.0015 (5)	0.0016 (4)
C8	0.0208 (6)	0.0204 (6)	0.0142 (6)	−0.0004 (5)	0.0004 (5)	−0.0010 (5)
C9	0.0269 (7)	0.0285 (7)	0.0161 (6)	0.0002 (6)	0.0050 (5)	−0.0006 (5)
C10	0.0307 (7)	0.0287 (7)	0.0196 (7)	−0.0019 (6)	0.0054 (6)	0.0078 (5)
C11	0.0258 (7)	0.0218 (6)	0.0248 (7)	−0.0045 (5)	0.0045 (6)	0.0046 (5)
C12	0.0224 (6)	0.0172 (6)	0.0196 (6)	−0.0016 (5)	0.0031 (5)	0.0008 (5)
N2	0.0192 (6)	0.0197 (5)	0.0279 (6)	−0.0004 (4)	0.0000 (5)	−0.0013 (5)
C13	0.0151 (6)	0.0149 (6)	0.0226 (6)	−0.0022 (5)	0.0061 (5)	−0.0035 (5)
S1	0.02052 (18)	0.02481 (18)	0.02222 (18)	0.00004 (12)	0.00086 (13)	0.00398 (12)

### Geometric parameters (Å, °)

**Table:**

N1—C7	1.5060 (16)	C6—H6B	0.9900
N1—C1	1.5132 (15)	C7—C12	1.5237 (17)
N1—H1A	0.926 (17)	C7—C8	1.5280 (17)
N1—H1B	0.901 (18)	C7—H7	1.0000
C1—C6	1.5216 (17)	C8—C9	1.5304 (18)
C1—C2	1.5226 (17)	C8—H8A	0.9900
C1—H1	1.0000	C8—H8B	0.9900
C2—C3	1.5301 (17)	C9—C10	1.528 (2)
C2—H2A	0.9900	C9—H9A	0.9900
C2—H2B	0.9900	C9—H9B	0.9900
C3—C4	1.5244 (18)	C10—C11	1.526 (2)
C3—H3A	0.9900	C10—H10A	0.9900
C3—H3B	0.9900	C10—H10B	0.9900
C4—C5	1.5243 (18)	C11—C12	1.5304 (18)
C4—H4A	0.9900	C11—H11A	0.9900
C4—H4B	0.9900	C11—H11B	0.9900
C5—C6	1.5317 (17)	C12—H12A	0.9900
C5—H5A	0.9900	C12—H12B	0.9900
C5—H5B	0.9900	N2—C13	1.1676 (18)
C6—H6A	0.9900	C13—S1	1.6448 (14)
			
C7—N1—C1	117.23 (9)	C5—C6—H6B	109.5
C7—N1—H1A	107.9 (10)	H6A—C6—H6B	108.1
C1—N1—H1A	106.6 (10)	N1—C7—C12	110.47 (10)
C7—N1—H1B	109.4 (11)	N1—C7—C8	108.69 (10)
C1—N1—H1B	106.6 (11)	C12—C7—C8	111.48 (10)
H1A—N1—H1B	108.8 (15)	N1—C7—H7	108.7
N1—C1—C6	110.54 (10)	C12—C7—H7	108.7
N1—C1—C2	107.84 (10)	C8—C7—H7	108.7
C6—C1—C2	112.16 (10)	C7—C8—C9	109.85 (11)
N1—C1—H1	108.7	C7—C8—H8A	109.7
C6—C1—H1	108.7	C9—C8—H8A	109.7
C2—C1—H1	108.7	C7—C8—H8B	109.7
C1—C2—C3	110.87 (10)	C9—C8—H8B	109.7
C1—C2—H2A	109.5	H8A—C8—H8B	108.2
C3—C2—H2A	109.5	C10—C9—C8	110.90 (11)
C1—C2—H2B	109.5	C10—C9—H9A	109.5
C3—C2—H2B	109.5	C8—C9—H9A	109.5
H2A—C2—H2B	108.1	C10—C9—H9B	109.5
C4—C3—C2	111.02 (10)	C8—C9—H9B	109.5
C4—C3—H3A	109.4	H9A—C9—H9B	108.0
C2—C3—H3A	109.4	C11—C10—C9	110.85 (11)
C4—C3—H3B	109.4	C11—C10—H10A	109.5
C2—C3—H3B	109.4	C9—C10—H10A	109.5
H3A—C3—H3B	108.0	C11—C10—H10B	109.5
C5—C4—C3	110.46 (11)	C9—C10—H10B	109.5
C5—C4—H4A	109.6	H10A—C10—H10B	108.1
C3—C4—H4A	109.6	C10—C11—C12	111.70 (11)
C5—C4—H4B	109.6	C10—C11—H11A	109.3
C3—C4—H4B	109.6	C12—C11—H11A	109.3
H4A—C4—H4B	108.1	C10—C11—H11B	109.3
C4—C5—C6	111.65 (11)	C12—C11—H11B	109.3
C4—C5—H5A	109.3	H11A—C11—H11B	107.9
C6—C5—H5A	109.3	C7—C12—C11	110.47 (11)
C4—C5—H5B	109.3	C7—C12—H12A	109.6
C6—C5—H5B	109.3	C11—C12—H12A	109.6
H5A—C5—H5B	108.0	C7—C12—H12B	109.6
C1—C6—C5	110.73 (10)	C11—C12—H12B	109.6
C1—C6—H6A	109.5	H12A—C12—H12B	108.1
C5—C6—H6A	109.5	N2—C13—S1	178.68 (12)
C1—C6—H6B	109.5		
			
C7—N1—C1—C6	56.44 (13)	C1—N1—C7—C12	60.50 (14)
C7—N1—C1—C2	179.38 (10)	C1—N1—C7—C8	−176.89 (10)
N1—C1—C2—C3	−176.97 (10)	N1—C7—C8—C9	−179.73 (10)
C6—C1—C2—C3	−55.03 (13)	C12—C7—C8—C9	−57.74 (14)
C1—C2—C3—C4	56.09 (14)	C7—C8—C9—C10	57.46 (14)
C2—C3—C4—C5	−56.87 (15)	C8—C9—C10—C11	−56.47 (15)
C3—C4—C5—C6	56.52 (15)	C9—C10—C11—C12	55.21 (16)
N1—C1—C6—C5	174.64 (10)	N1—C7—C12—C11	177.35 (10)
C2—C1—C6—C5	54.26 (13)	C8—C7—C12—C11	56.39 (14)
C4—C5—C6—C1	−54.99 (14)	C10—C11—C12—C7	−54.95 (15)

### Hydrogen-bond geometry (Å, °)

**Table:**

*D*—H···*A*	*D*—H	H···*A*	*D*···*A*	*D*—H···*A*
N1—H1B···N2	0.901 (18)	1.986 (19)	2.8811 (17)	172.8 (16)
N1—H1A···S1^i^	0.926 (17)	2.440 (17)	3.3610 (13)	172.8 (13)

Symmetry codes: (i) *x*+1/2, −*y*+1/2, −*z*+1.
